# Rectal colonization is predictive for surgical site infections with multidrug-resistant bacteria in abdominal surgery

**DOI:** 10.1007/s00423-023-02961-x

**Published:** 2023-06-10

**Authors:** Matthias Mehdorn, Susanne Kolbe-Busch, Norman Lippmann, Yusef Moulla, Uwe Scheuermann, Boris Jansen-Winkeln, Iris F. Chaberny, Ines Gockel, Woubet Tefera Kassahun

**Affiliations:** 1 Department of Visceral, Transplant, Thoracic and Vascular Surgery, University Hospital of Leipzig, Liebigstraße 20, 04103 Leipzig, Germany; 2 Institute of Hygiene, Hospital Epidemiology and Environmental Medicine, University Hospital of Leipzig, Leipzig, Germany; 3 Institute for Medical Microbiology and Virology, University Hospital of Leipzig, Leipzig, Germany; 4 Department of General, Visceral and Oncological Surgery, Klinikum St. Georg, Leipzig, Germany

**Keywords:** Multidrug-resistant organisms, Superficial surgical site infections, Rectal colonization, Abdominal surgery, Visceral surgery

## Abstract

**Purpose:**

Superficial surgical site infections (SSI) are a common complication after abdominal surgery. Additionally, multidrug-resistant organisms (MDRO) have shown an increasing spread in recent years with a growing importance for health care. As there is varying evidence on the importance of MDRO in different surgical fields and countries as causative agents of SSI, we report our findings of MDRO-caused SSI.

**Methods:**

We assembled an institutional wound register spanning the years 2015–2018 including all patients with abdominal surgery and SSI only, including demographics, procedure-related data, microbiological data from screenings, and body fluid samples. The cohort was examined for the frequency of different MDRO in screenings, body fluids, and wound swabs and assessed for risk factors for MDRO-positive SSI.

**Results:**

A total of 138 out of 494 patients in the register were positive for MDRO, and of those, 61 had an MDRO isolated from their wound, mainly multidrug-resistant *Enterobacterales* (58.1%) followed by vancomycin-resistant *Enterococcus* spp. (19.7%). As 73.2% of all MDRO-carrying patients had positive rectal swabs, rectal colonization could be identified as the main risk factor for an SSI caused by a MDRO with an odds ratio (OR) of 4.407 (95% CI 1.782–10.896, *p* = 0.001). Secondly, a postoperative ICU stay was also associated with an MDRO-positive SSI (OR 3.73; 95% CI 1.397–9.982; *p* = 0.009).

**Conclusion:**

The rectal colonization status with MDRO should be taken into account in abdominal surgery regarding SSI prevention strategies.

**Trial registration** Retrospectively registered in the German register for clinical trials (DRKS) 19th December 2019, registration number DRKS00019058.

## Introduction

Superficial surgical site infections (SSSI) carry a major morbidity burden after abdominal surgery. The most common treatment of these wounds is open wound treatment with daily dressing changes in order to evacuate wound secretion and to ensure clean wound granulation. Furthermore, modern wound dressings are designed to absorb bacteria that are part of the wound biofilm which evolves during wound healing. These bacteria differ based on the acuity of the wound [1, 2], potential wound contamination by previous surgical interventions, and regional bacterial prevalence patterns [3]. In accordance with these local prevalence patterns, the frequency of different multidrug-resistant organisms (MDRO) varies [4-6]. There are reports from countries in Asia, where MDRO bacteria are very common in wound secretion samples with methicillin-resistant staphylococcus aureus (MRSA) rates as high as 60%, MDR *Escherichia coli* with 80% and MDR *Klebsiella pneumoniae* with 83.3% [7]. Carbapenem resistance is also of concern not only in developing countries [5] but also worldwide, as high numbers of deaths are attributable to antimicrobial resistance patterns [8]. Multiple studies have proven that antibiotic stewardship programs significantly reduce the incidence of infection and colonization with antibiotic-resistant bacteria [8-10]. Nonetheless, this problem might also have other causes than the unrestricted use of antibiotics within the health care system. It has been shown that high levels of antibiotics are present in rivers and wastewater depots around Indian drug factories, and subsequently, a lot of MDRO strains could be isolated from those waters, including carbapenemase-producing strains [10].

Apart from standard skin flora, MDRO may not only occur as colonizing commensals, but also as pathogens causing SSSI. The connection between pre-existing MRSA colonization and post-operative SSI (MRSA), either in soft tissue infections [11] or in SSI after liver surgery [12], has been shown. Nosocomial infections with MDRO seem to be associated with a prolonged hospital stay and more severe complications [13]. Therefore, it has been stressed that hygiene measures are mandatory to prevent transmission of MDRO in elderly patients [14]. There have been reports, however, that omitting contact precautions would not lead to an increased rate of infections by MRSA or vancomycin-resistant *enterococci* (VRE) [15].

As SSSI may be colonized with or caused by MDRO, they also might serve as a reservoir for the spread of these bacteria in patients that are at risk of developing severe infectious complications in the postoperative course. Current evidence on the frequency of MDRO in SSSI after abdominal surgery is scarce for Germany.

Therefore, the aim of this study was to elucidate the type and frequency of MDRO in patients with SSSI and subsequent SSSI caused by MDRO in a German tertiary referral hospital after abdominal surgery. Furthermore, this study elaborates on the importance of the surgical site infection as a potential reservoir for MDRO and describes potential risk factors for the infection of the wound with MDRO.

## Methods

Our study has been reviewed and approved by the ethics review board of the University of Leipzig (419/18-ek) and has been registered in the German register for clinical trials (DRKS).

### Patient selection

Our clinical wound register was assembled including patients that had been diagnosed with superficial (epifascial) surgical site infections after abdominal surgery according to the American Center for Disease Control and Prevention (CDC) criteria [16] between the 1st of January 2015 through the end of December 2018, including prospectively collected patient data from the electronic patient chart. Data were collected prospectively, and the analysis was carried out retrospectively. We included information of patients with SSSI, but excluded patients who suffered from organ space abscesses requiring radiologically guided drainage only or who had to undergo reoperation. All patient-related data (age, sex, diagnosis, surgical procedure at admission, comorbidities) were assembled. Furthermore, data about the postoperative course, including postoperative complications and therapeutic abnormalities such as revision surgery of the index procedure, were included. All treatment-related parameters refer to the index hospitalization period and not to previous or following ones. Additionally, data regarding microbiological cultures from wound swabs were obtained and have been presented previously [3]. With respect to the current study, data about MDRO—either isolated from routine screenings or from cultures of wound swabs—were collected.

MDRO were divided as follows: MRSA was defined as *Staphylococcus aureus* resistant against oxacillin and VRE is defined as *Enterococcus faecium* or *Enterococcus faecalis* resistant against vancomycin. Gram-negative organisms, that are *Enterobacterales*, *Pseudomonas aeruginosa*, and *Acinetobacter baumanni*, were considered multidrug resistant according to the recommendations of the German Commission of Hospital Hygiene and Infection Prevention (KRINKO) [17]. Resistance to three or four out of four agents (piperacillin, ciprofloxacin, carbapenems, and either cefotaxime or ceftazidime) was defined as multidrug resistance.

It was recorded, if those MDRO were either already known or detected within the first 2 days after admission, if the pathogens were acquired during hospital stay, or if these MDRO were isolated from the surgical wound or from other sites.

### Screening for MDR bacteria

Risk factors for potential colonization with MDRO are surveyed upon admission for each patient, and MDRO screening is performed on a risk-based strategy according to the recommendations of the German KRINKO [17-19] by a standardized questionnaire. MRSA screening is performed via PCR or surveillance culture in case of known history of MRSA colonization or if a patient is transferred from another hospital or a nursing facility, received antibiotic medication within the last 12 months, required hemodialysis, or if the patient is to be treated on the intensive care unit (ICU). Screening for MDR gram-negative (MDRGN) from culture of rectal and/or stool swabs is carried out if the patient has a known colonization, planned or received transplantation of bone marrow or solid organs, received antibiotic treatment within the last 12 months, or is to be treated on the ICU. A screening for VRE is carried out in those patients who were previously colonized with VRE, who were transferred from another hospital, or who were treated as in-patients within the last 12 months. Additionally, all those screenings will be performed in those patients who have resided in countries with known high MDR prevalence.

In order to determine the type and frequency of MDRO in the department’s patient population, the screening tests for MRSA, MDRGN, and VRE were evaluated using the database of the Institute of Hygiene, Hospital Epidemiology, and Environmental Medicine. A positivity rate was calculated from the case-related total number of screening tests and the number of positive findings. Unfortunately, routine screening of VRE started only in 2018, so the rates of the years 2015–2017 were very low. Therefore, the VRE positivity rate could not be related to the whole study period, and interpretation with regard to VRE isolates from wounds was limited.

### Determination of resistance patterns

All microbiologic samples were tested in the clinical microbiology laboratory of the Institute of Medical Microbiology and Virology of the University of Leipzig Medical Center.

### Screening for MDRO

For screening, we use the eSwab kit for aerobic, anaerobic, and fastidious bacteria (Copan, Brescia, Italy). Nasal and oropharyngeal swabs were used for MRSA screening, as rectal swabs or stool specimens were used for VRE and MDRGN screenings.

MRSA testing was performed by PCR using the Xpert® MRSA NxG (Cepheid, Sunnyvale, USA) kit. The detection of carbapenemases was carried out with the Xpert® Carba-R PCR kit (Cepheid, Sunnyvale, USA), and vancomycin resistances were detected by the Xpert® vanA/vanB (Cepheid, Sunnyvale, USA) PCR.

### Species identification and antimicrobial susceptibility testing

Diagnostic isolates were cultivated, and species identification and antimicrobial susceptibility testing of isolates were performed according to standardized procedures. Organisms were categorized as susceptible, intermediate, or resistant to the antimicrobial agent in question according to the European Committee of Antibiotic Susceptibility Testing (EUCAST) breakpoints [20].

### Statistical analysis

All data were collected using Microsoft Excel (Microsoft, Munich, Germany), and SPSS 27 (IBM statistics, Ehningen, Germany) was used for statistical analysis.

All dichotomous variables are expressed using relative frequencies (absolute number). Continuous variables instead were expressed as median (± standard deviation = SD) after testing for normal distribution. To compare continuous variables, the unpaired *t*-test was used.

To compare dichotomous variables, we used cross-tab calculation with subsequent chi^2^-test. A *p* = 0.05 was considered to be statistically significant.

For further analysis of risk factors, we performed a multivariate analysis, using the stepwise binary logistic regression model, again considering *p* = 0.05 to be statistically significant. We entered all risk factors from the univariate analysis into the multivariate analysis that had a *p* < 0.1.

Odds ratios (OR) were calculated with the respective 95% confidence interval (95% CI).

## Results

Between January 2015 and December 2018, a total of 494 patients developed SSSI and were included in our institutional wound register. Of these, 138 (80 males and 58 females) were identified as having a MDRO either by screening or by routine microbiological testing from wound swabs or other body fluids. The other patient-specific data can be deduced from Table 1. Most common procedures were colorectal (23.4%), liver surgery (16.3%), small bowel resections (12.1%), and explorative laparotomies without any resection (12.1%).

**Table 1:** Comparison of patient characteristics between the SSSI group and MDRO patients. Values are given as percentages (absolute number) or mean (± standard deviation). The *p*-values of the chi^2^ test are marked with an asterisk if *p* < 0.05 or two asterisks if *p* < 0.01

	No MDRO (*n* = 356)	MDRO (*n* = 138)	*p*-value
Age	62.67 ± 14.66	61.15 ± 14.98	0.305
ICU Stay (*n* = 373)	73.2 (260)	81.9 (113)	0.047*
Chronic inflammatory disease (*n* = 53)	10.1 (36)	12.3 (17)	0.517
Past or present malignant Disease (*n* = 289)	59.0 (210)	57.2 (79)	0.76
Past or present chemotherapy (*n* = 119)	24.7 (88)	22.5 (31)	0.640
Immunosuppressants (*n* = 91)	15.7 (56)	25.4 (35)	0.019*
Diabetes mellitus (*n* = 125)	23.0 (82)	31.2 (43)	0.066
Chronic renal failure (*n* = 265)	52.8 (188)	55.8 (77)	0.615
Liver Cirrhosis (*n* = 59)	10.4 (37)	15.9 (22)	0.091
Burst abdomen (*n* = 111)	19.4 (69)	30.4 (42)	0.011*
Emergency (*n* = 181)	35.4 (126)	39.9 (55)	0.405
V.A.C. therapy (*n* = 262)	48.9 (174)	63.8 (88)	0.004**
Surgical Revision (*n* = 226)	39.6 (141)	61.6 (85)	< 0.001**
Antibiotic therapy (*n* = 231)	64.3 (128)	77.4 (103)	0.011*
LOS	26.60 ± 15.63	38.91 ± 21.323	< 0.001**
Acute renal failure	32.2 (114)	45.7 (63)	0.006**
Postoperative Pneumonia	8.1 (29)	6.5 (9)	0.707

*ICU* intensive care unit, *BMI* body mass index, *LOS* length of stay

Overall, 6713 patients received an abdominal operation, and 494 of those developed an SSSI and thus were eligible for inclusion in the wound register in that period. As 138 patients were diagnosed with MDRO colonization or infection, the positivity rate is at 27.9% for an MDRO in any localization and 11.8% for patients with MDRO-positive SSI.

### Screening for MDRO

During the period of the wound register, 10,583 screenings were performed for MDR bacteria (*n* = 5699) and MRSA (*n* = 4884) following the previously described questionnaires. Routine VRE screening started in 2018 and included 795 patients. Thus, the interpretation of the screening results could only refer to the single year instead of the whole study period.

In the entire University of Leipzig Medical Center, approximately 60% and 46% of all patients were screened for MRSA and MDRGN respectively.

In patients of the surgical department, the positivity rate for MDRGN increased from 4.2% in 2015 to 8.1% in 2018, and for MRSA, it decreased from 1.7 to 0.8% in the same period. Screening for VRE was positive in 5.7% in 2018.

Of those 138 MDRO-positive patients, 62 (44.9%) had a known history of MDRO colonization and therefore were screened per protocol in all sites. In 76 (55.1%) patients, a MDRO was diagnosed during the hospital stay. The screening for those patients at admission was as follows: Forty-six (60.5%) received complete screening for MDRGN, MRSA, and VRE; 9 (11.8%) only rectal swabs for MDRGN; and 6 (7.9%) only MRSA screening. Thus, 15 patients (19.7%) did not show any risk factors of being MDRO carrier and were not screened.

Table 2 shows the sites from which positive samples could be yielded. The patients with a known history of MDRO carriage, rectal, and nasopharyngeal positivity were results from screening tests at admission confirming prior known carriage. Positivity in body fluids resulted from samples taken after the index procedure. For patients with diagnosis of MDRO carriage or infection during hospitalization, all samples were obtained after the index procedure. Furthermore, of those patients screened according to the protocols mentioned before, the conversion rate of rectal colonization was as follows: 27 out of 46 completely screened patients, and 8 out of 9 of the rectally screened patients developed rectal MDRO carriage.

**Table 2:** Sampling sites that yielded MDRO isolates. Data is given as relative frequency (absolute number). The *p*-values refer to the chi^2^-test

	Overall Cohort (*n* = 138)	Known history of MDRO (*n* = 62)	Diagnosis of MDRO during stay (*n* = 76)	*p*-value
Rectal/stool	73.2 (101)	83.9 (52)	64.5 (49)	0.012*
Urine	18.1 (25)	19.4 (12)	17.1 (13)	0.825
Nose/throat	6.5 (9)	11.3 (7)	2.6 (2)	0.078
Wound	44.2 (61)	43.5 (27)	44.7 (34)	1
Intraabdominal	17.4 (24)	12.9 (8)	21.1 (16)	0.261
Sputum	3 (0.6)	0	3.9 (3)	0.252
Bloodstream	8.0 (11)	6.6 (4)	9.2 (7)	0.754
Bodyfluid	2.9 (4)	1.6 (1)	3.9 (3)	0.627

### MDRO colonizing patients

A total of 171 different MDRO could be isolated from those 138 patients, resulting in a median of one MDRO per patient.

The most common isolated bacterial species from screening and body fluids was MDR *Escherichia coli* (47.1%), followed by vancomycin (vanA) resistant *Enterococcus faecium* (23.9%) and MDR *Klebsiella pneumoniae* (13.0%). Grouping the MDRO according to the EUCAST groups, the most frequently isolated bacteria were MDR *Enterobacterales*. Further results are listed in Table 3.

**Table 3:** Isolated MDRO according to EUCAST groups. Values are given as percentages (absolute number). The first column depicts all patients with MDRO, including those patients with more than one MDRO. The second column divides all isolated MDRO, and the third column only includes MDRO isolated from SSSI

	Patients with MDRO (*n* = 138)	All MDRO (*n* = 160)	MDRO in wound (*n* = 61)
MDR Enterobacterales	66.7 (92)	57.5 (92)	58.1 (36)
VRE	30.4 (42)	26.3 (42)	19.7 (12)
MDR *Pseudomonas* spp.	8.7 (12)	7.5 (12)	11.3 (7)
MRSA	8 (11)	6.9 (11)	8.1 (5)
MDR *Acinetobacter* spp.	2.2 (3)	1.9 (3)	1.6 (1)

The most common positive sampling site was rectal/stool (73.2%) followed by wound (44.2%), urine (18.1%), and intraabdominal (17.3%). In our patients, 44.2% were colonized in one site only, 44% in two, and 12% in 3 or more sites. Only nine patients had a nasopharyngeal colonization with MRSA.

#### Risk factors for MDROs in hospitalized patients

We analyzed the risk factors of all patients carrying MDROs and compared them to those patients in our wound register that were never colonized by or infected with a MDRO. The base data is visible in Table 1. The univariate analysis, using the chi^2^ test showed immunosuppressive medication (*p* = 0.019) as the only significant comorbid risk factor for MDRO carriage during the course of inpatient treatment. Diabetes mellitus (*p* = 0.109) and liver cirrhosis (*p* = 0.113) failed to reach significance levels. Of the treatment-related parameters during the hospitalization period after the index procedure, necessity for surgical revision (*p* < 0.001), length of stay (*p* < 0.001), vacuum-assisted closure (V.A.C.) therapy (*p* = 0.004), burst abdomen (*p* = 0.011), antibiotic therapy for infection (*p* = 0.011), and ICU stay (*p* = 0.047) were significantly associated with the colonization of an MDRO carriage. The multivariate regression analysis, using all those aforementioned parameters with *p* < 0.1, only considered immunosuppressive medication (OR 1.995; 95% CI 1.111–3.584, *p* = 0.021) and length of stay (OR 1.026, 95% CI 1.014–1.039, *p* < 0.001) as significant risk factors for the presence of MDRO on patients.

### MDROs detected in wounds

#### MDRO spectrum found in wound swabs

**Fig. 1:**
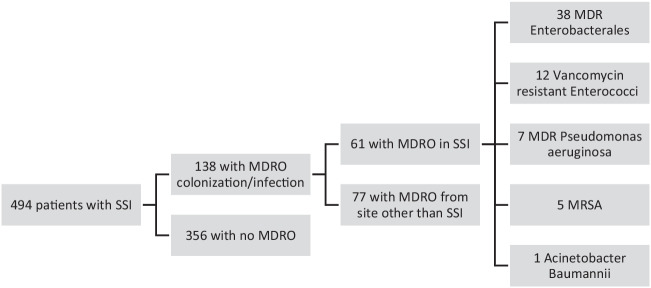
Displaying the patients from the wound register with MDRO and those with MDRO-caused SSI. The specific bacteria are listed in the last column

In 61 patients, MDRO could be isolated from their SSSI. The isolates from wounds can be deduced from Fig. 1 and Table 3.

Ten patients had the wound as their only site of MDRO detection without any other previously known colonization. The detected strains were five MDR *P. aeruginosa*, two MDR *K. pneumoniae*, two MDR *E. coli*, and two VRE (vanA) *E. faecium* but no MRSA. These ten patients represent 16.4% of all patients with MDRO in the SSSI.

#### Risk factors associated with MDRO-positive SSSI

Further analyses were performed on those patients with an MDRO detected in any sampling site and compared them with patients with MDRO-positive SSSI in order to find possible risk factors for the wound infections with MDRO. Only chronic inflammatory diseases were a significant risk factor for MDRO infection in the wound (*p* = 0.034). Data are listed in Table 4. Intestinal resection occurred in 203 patients of the whole cohort, but no significant correlation could be found for intestinal resection and MDRO-positive SSI in patients with rectal colonization (Rho = 0.177; *p* = 0.77).

**Table 4:** Characteristics of patients with MDRO either with or without MDRO-positive SSSI. The last paragraph depicts continuous variables of patients with SSI ± MDRO colonization/Infection. Values are given as percentages (absolute number) or mean (± standard deviation). The *p*-values are marked with an asterisk if < 0.05 or two asterisks if < 0.01

	MDRO from site other than SSI (*n* = 77)	MDRO in SSI (*n* = 61)	*p*-value
Chronic inflammatory disease (*n* = 17)	6.5 (5)	19.7 (12)	0.034*
Past or present malignant disease (*n* = 79)	59.7 (46)	54.1 (33)	0.604
Past or present chemotherapy (*n* = 31)	20.8 (16)	24.6 (15)	0.682
Immunosuppressants (*n* = 35)	26.0 (20)	24.6 (15)	1
Diabetes mellitus (*n* = 43)	37.7 (29)	23.0 (14)	0.068
Chronic renal failure	61 (47)	49.2 (30)	0.173
Liver Cirrhosis (*n* = 22)	19.5 (15)	11.5 (7)	0.246
Burst abdomen (*n* = 42)	24.7 (19)	37.7 (23)	0.136
Emergency (*n* = 55)	41.6 (32)	37.7 (23)	0.727
V.A.C. therapy (*n* = 88)	59.7 (46)	68.9 (42)	0.29
Surgical Revision (*n* = 85)	58.4 (45)	65.6 (40)	0.481
Antibiotic therapy (*n* = 103)	76.4 (55)	78.7 (48)	0.836
ICU stay (*n* = 113)	89.6 (69)	72.1 (44)	0.013*
Bloodstream (*n* = 11)	13.0 (10)	1.6 (1)	0.023*
Intraabdominal (*n* = 24)	15.6(12)	19.7 (12)	0.652
Sputum (*n* = 3)	3.9 (3)	0	0.255
Rectal/stool (*n* = 101)	83.1 (64)	60.7 (37)	0.004**
Urine (*n* = 25)	16.9 (13)	19.7 (12)	0.824
Nose/throat (*n* = 9)	5.2 (4)	8.2 (5)	0.508
Age	58.6 ± 16.01	63.17 ± 13.88	0.075
BMI	25.96 ± 6.49	26.61 ± 5.71	0.546
ICU LOS	5.78 ± 8.13	10.06 ± 11.01	0.02*
LOS	38.39 ± 20.92	39.32 ± 21.76	0.80

*ICU* intensive care unit, *BMI* body mass index, *LOS* length of stay

The parameters of the postoperative course only elucidated a post-operative ICU stay (*p* = 0.013) as significantly associated with an MDRO-positive SSSI. Even the previously significant risk factors for the colonization with a MDRO (burst abdomen, antibiotic therapy, V.A.C. therapy, and surgical revision) failed to reach significance levels. Rectal colonization was significantly associated with a MDRO wound infection (*p* = 0.004), as well as blood stream infection with MDRO (*p* = 0.023). The relative frequencies of rectal colonization, blood stream infection, and ICU stay were lower in the MDRO SSI group. But compared to other parameters, the incidence in the MDRO SSSI group was comparably higher. Only eleven patients had a blood stream infection with an MDRO, and of those, only one patient a MDRO-positive SSSI.

The multivariate regression model, setting MDRO-positive SSSI as a reference parameter, clarified that for a SSSI caused by MDRO, the factors blood stream infection (OR 10.404; 95% CI 1.165–92.933, *p* = 0.036), rectal/stool colonization (OR 4.407; 95% CI 1.782–10.896, *p* = 0.001), and ICU stay (OR 3.734; 95% CI 1.397–9.982, *p* = 0.009) were the important and significant risk factors.

### Antibiotic use in patients with MDRO in SSSI

As stressed during the risk factor analysis, post-operative antibiotic treatment itself was an independent risk factor for MDRO carriage, but could not prove its importance in the multivariate analysis as 98 (71.0%) of the MDRO colonized patients received an antibiotic therapy. Although, in the univariate analysis of patients from the MDRO subgroup with VRE, antibiotic therapy was significantly associated with the presence of VRE but not with other types of MDRO.

Of the 61 patients with MDRO-positive SSSI, 45 received an antibiotic treatment for various causes. The antibiotic regime respected the MDRO status in 29 (64.4%) of the patients.

## Discussion

We analyzed the type and frequency of MDRO in our tertiary referral hospital of patients with SSSI following abdominal surgery. Our study shows the frequency of MDR bacteria and their subsequent importance for SSSI. The most common causative bacteria for SSSI were MDR *Enterobacterales* and VRE. But, as only 12.3% of the SSSI were caused by MDRO, their importance for wound infections remains limited. Nonetheless, rectal colonization with MDRO was shown to be a significant risk factor for a MDRO-positive SSSI.

### Screening for MDRO

Knowing the patient’s preoperative colonization status with MDR bacteria has proven beneficial in different surgical specialties and indications, linking MRSA to post-operative soft tissue infections [21] or SSI after hepatobiliary surgery [12] and MDRGN bacteria in obstructive bile duct pathologies requiring pancreatic head resection [22]. But when screening patients preoperatively for certain MDR bacteria, one would have to bear in mind their importance for post-operative SSI formation. We previously published the spectrum of bacterial isolates from the SSSI in our hospital, mainly consisting of gram-negative bacteria and *Enterococcus* spp. instead of *S. aureus* [3]. Therefore, the need for MRSA screening in abdominal surgery seems weak which is supported by data from the USA and Switzerland [23]. Hence, it seems adequate to perform screenings in a problem-oriented manner.

Our department’s screening positivity rates displayed increasing rates of MDR colonization over the time period between 2015 and 2018, whereas the MRSA positivity rates decreased over the study period (data not shown). Patients who developed an SSSI showed a steady rate of MDRO colonization or infection with no clear trend over time. The positivity rate of VRE screening for the whole department in 2018 was at about 5.7% and thus was at a slightly lower rate than that of MDRGN bacteria (8.1% in 2018), which is still remarkably higher than MRSA (0.8% in 2018). But still, it was much higher than the VRE rate reported from Belgium of 0.6% [14]. Maybe this correlates with the increase in VRE occurrence in German hospitals during the last decades [6]. Therefore, screening considerations should also include geographical factors.

### Risk factors for MDRO in SSSI

Positivity rates of MDR screenings on the one hand and MDR isolates from body fluids on the other hand differ mightily. The SSSI cohort showed a five-fold higher positivity rate of MDRO carriage compared to the department’s screening results. Although, already 44.9% of the cohort had a known history of MDRO carriage and only 55.1% developed or acquired MDRO carriage during the hospitalization. Whereas less than half of the patients carrying MDRO were diagnosed with an SSSI caused by an MDRO. Those numbers are still considerable, but patients with an SSSI are a selected cohort with a longer hospital stay and several comorbid conditions as risk factors for the presence of an MDRO. As permanent surveillance of MDRO rates and time-dependent coincidences in departments and wards is carried out by the Institute of Hygiene, Hospital Epidemiology, and Environmental Medicine, a lack in hygiene measures as causative for this rate can be excluded. The same applies for the screening questionnaires that follow German guidelines. We described immunosuppressive medication as patient immanent significant risk factor for the carriage of an MDRO in the SSSI collective, but also the length of stay. As we know, patients with SSI endure longer hospital stay due to additional therapeutic efforts that might predispose for MDRO acquisition, i.e., antibiotic therapy. Hence, the longer they are hospitalized, the higher the risk of MDRO acquisition.

Rectal colonization and postoperative ICU stay were important risk factors considering the subgroup of patients with a wound infection with MDRO. These results seem very reasonable as in visceral surgery, the colon or the small bowel are often incised during the procedure, and subsequently, a bacterial spread may occur despite the fact that in our cohort, no significant correlation could be found between surgeries including bowel resections and MDRO-positive SSI with rectally isolated bacteria. Probably not only direct contamination during the surgery by opening the bowel but also bacterial spread through translocation from the intestine might play a role [24]. Thus, surgical teams should pay a significant attention to maintain optimal sterile working conditions, i.e., by considering preoperative bowel preparation [25], using special tools or techniques intraoperatively [26] or antiseptic wound irrigation at the end of surgery [27].

### MDRO isolates from SSSI

#### MRSA

*Enterobacterales* and *Enterococcus* spp. are the most frequent strains in SSSI after abdominal surgery [3]. Our results have underlined the subordinate role of S. aureus in SSI formation in abdominal surgery. In the present study, only eleven patients carried MRSA, with nine positive swabs in nasopharyngeal screenings, resulting in five MRSA-positive SSSI. All patients with MRSA-positive SSSI had nasopharyngeal colonization. Although 5 (55.6%) of the patients with a positive nasopharyngeal screening developed MRSA-positive SSSI, the total number of MRSA-positive patients remains comparably limited. Furthermore, patients with *S. aureus* infections were mostly after abdominal wall reconstruction as has been shown in literature, too [28]. Those bacteria are of bigger importance for SSI after operations that do not incise the gastrointestinal tract.

If you consider screening to guide hospital hygiene measures, one would need to take into account patients’ countries of origin, temporary residence, and previous hospital locations if patients are directly transferred from another hospital or even their profession [29, 30]. But also, the patients’ profession might hint at MDRO carriage, as nasopharyngeal MRSA carriage was present in 39.4% in Ukrainian healthcare workers of otorhinolaryngology [31]. In our collective, only 5 out of 30 *S. aureus* strains (16.7%) isolated from the SSSI were MRSA. This value is even lower than in most parts of the world, where positivity rates between 27.7% in India and 86% in Egypt [5, 13, 34,32-] have been reported from wound isolates.

#### MDRGN

As mentioned before, SSSI after abdominal surgery are mostly caused by gram-negative bacteria and *Enterococcus* spp. Therefore, we should further focus on gram-negative bacteria such as *E. coli* or *K. pneumoniae* but also *Enterococcus* spp., bacteria that are part of the gut microbiome.

MDRGN bacteria pose a challenge around the world [35-39] as well as in our institution. We could show that 66.7% of all isolated resistant strains were MDR *Enterobacterales*. Furthermore, 59% of the resistant bacteria isolated from wound swabs were *Enterobacterales*. This is of special interest regarding preoperative single-dose antibiotic prophylaxis. German recommendations include a 2nd generation of cephalosporine in most of the operations of the gastrointestinal tract, but MDRGN bacteria would be resistant to the single-dose antibiotic. Furthermore, other bacteria that expectedly would be susceptible to a 2nd generation cephalosporin showed resistance in almost 50% in the wound isolates from SSI after trauma surgery in our institution [40]. This stresses the point that one should know the locally prevailing resistance patterns of bacterial strains and patient’s colonization status via screening, as those patterns may change over time, especially with regard to choosing the most suitable preoperative antibiotic prophylaxis. The lack of recent evidence on local resistance patterns and colonizations in our region was one of the major key points to conduct this analysis.

Besides resistance to cephalosporines, an even more threatening resistance pattern is carbapenemase production as the treatment options of those bacteria are very limited. In our cohort, only one patient had a wound infection with a carbapenem-resistant *P. aeruginosa*. Thus, these resistance patterns did not play a significant role in our cohort and in clinical routine. Unfortunately, in other settings, more alarming prevalences have been reported [5]: 100% of *Acinetobacter baumanii* and 70% of *K. pneumoniae* were CRE. In order to prevent worsening of the bacterial resistance patterns, the most effective measure stressed by a lot of surveillance studies would be the implementation of a strict antibiotic stewardship [9, 10, 35, 36].

#### VRE

Besides MDRGN, also, *Enterococcus* spp. are of concern as causative agents of SSSI after abdominal surgery. In Germany, increasing incidences of VRE have been reported in healthcare-associated infections with different strains being prevalent in distinct episodes [6], and Germany was apparently one of the countries with the most pronounced increase of VRE [18]. Subsequently, a risk-based VRE screening was introduced in our institution and yielded a positivity rate of 5.7% in 2018. There have been VRE outbreaks across Europe in recent years [41-43], and studies have published different screening schemes and laboratory methods that consequently revealed different carriage rates (0.4% up to 28.7%) [44-47].

The second most frequent group of MDRO in the SSSI were VRE (19.7%). In total, we had isolated 175 strains of *Enterococcus* spp. [3], which results in 6.9% of those being VRE. This would be less than in the USA [4] and Egypt [9] with 12% and 13%, respectively. Therefore, the difference between screening results and positivity rates in wound isolates was less pronounced for VRE than for the MDRGN. We have described repetitive surgery as the main risk factor for the presence of *Enterococcus* spp. in SSI previously [3] which was not triggered by the use of antibiotics. Nonetheless, the present analysis shows post-operative antibiotic use to treat postoperative infections as a risk factor for the presence of VRE.

### The wound as reservoir for MDRO

The reservoir of MDRO is different depending on the bacterial species. The gram-negative bacteria or *Enterococcus* spp. are typically found in the gastrointestinal tract. *S. aureus* can be a component of the nasopharyngeal flora or of the skin. The connection between preoperative colonization with MRSA or MDRGN and postoperative wound infection or organ space abscess has been established before [12, 22, 37]. As most of the SSSI showed either *Enterobacterales* or *Enterococcus* spp. in their wound cultures, subsequently, MDR *Enterobacterales* and VRE were the most common MDRO in the cultures. There was a statistical relation during risk—factor analysis between rectal colonization in screening tests and the postoperative samples from wound fluids. This becomes more obvious by the bare numbers that out of 77 patients with only one known MDRO from rectal swaps, the same MDRO could also be isolated from the wound in 47 of them. Only ten patients had the MDR bacteria cultivated solely from their wound swabs but from no other screened location. Half of those bacteria were MDR *P. aeruginosa*, a bacterial species with a well-known increased infection rate over recent years [48]. The antibiotic therapy was adapted to the resistance pattern in three of those patients, but it did not influence the antibiotic therapy in five of them. Reasons for this include a different suspected source of infection or later discovery of the MDRO, i.e., it was selected by the previous antibiotic regimen. Hence, the wound was the only location in 7.2% of the patients, which represents a small but important subgroup that should not be disregarded.

Once the MDRO is isolated from the wound, the patient will undergo contact precautions (CP), that demand an increasing amount of time for the medical staff. The ongoing discussion on the effectiveness of CP [15] is also fueled by the knowledge that patients under those measures are likely to feel stigma, perceive a lower quality of care, and feel isolated from their relatives, even beyond discharge [49]. There are hints that the extent of negative reception differs in accordance with the measures that also depend on the specific MDRO [39]. A problem, encountered in daily routine, is how to deal with a patient that had an MDRO isolated from the SSSI that meanwhile has healed. Once the patient is readmitted, quick screenings of the possible colonization sites (such as the stool) should be performed in order to liberate the patient from CP because the wound might have healed in the meantime. Especially since it is known that a loss of colonizing-resistant bacteria occurs with longer distance to index admission [50, 51].

### Limitations

Our study has several limitations that are inherent to its retrospective character. Unfortunately, routine VRE screening started only in 2018, which did not allow us to fully include it in the analysis. The message on VRE is therefore limited although it was the second most frequent MDRO in our group of SSSI patients. Furthermore, in our screening rates, patients with non-operative stays on our ward were also included. Probably, the collective of patients with a complicated post-operative course exhibit a higher risk of being colonized or suffering from an SSI by an MDRO. Hence, the results from the screening and the positivity rates in the SSI collective represent two different risk groups.

## Conclusion

In our cohort analysis of patients with SSSI after abdominal surgery, we clearly showed the importance of MDRO as bacteria causing SSSI and stress the risk factors of immunosuppressive medication and length of hospital stay for the colonization with an MDRO. Furthermore, in visceral surgery, rectal colonization was predictive for an SSSI caused by an MDRO. Only a few patients had their wounds as the primary reservoir for the MDRO, which still forms a relevant group. More targeted screening will help to predict and prevent possible infectious complications with MDRO in specific disciplines.

## Data Availability

The raw data of this study is accessible from the corresponding author upon reasonable request. It has not been stored in a publicly available data repository.
